# Oscillating dietary crude protein concentrations increase N retention of calves by affecting urea-N recycling and nitrogen metabolism of rumen bacteria and epithelium

**DOI:** 10.1371/journal.pone.0257417

**Published:** 2021-09-10

**Authors:** Ningning Zhang, Zhanwei Teng, Pengtao Li, Tong Fu, Hongxia Lian, Linfeng Wang, Tengyun Gao

**Affiliations:** College of Animal Science and Technology, Henan Agricultural University, Zhengzhou, Henan, China; University of Illinois, UNITED STATES

## Abstract

The purpose of this study was to investigate the effects of oscillating crude protein (CP) concentration diet on the nitrogen utilization efficiency (NUE) of calves and determine its mechanism. Twelve Holstein calves were assigned randomly into static protein diet (SP, 149 g/kg CP) and oscillating protein diet (OP, 125 and 173 g/kg CP diets oscillated at 2-d intervals) groups. After 60 days of feeding, the weights of total stomach, rumen and omasum tended to increase in calves fed OP. The apparent crude fat digestibility, NUE and energy metabolism also increased. In terms of urea-N kinetics evaluated by urea-^15^N^15^N isotope labeling method, the urea-N production and that entry to gastrointestinal tended to increase, and urea-N reused for anabolism increased significantly in calves fed OP during the low protein phase. These data indicate that urea-N recycling contributed to improving NUE when dietary protein concentration was low. In addition, the differentially expressed genes in rumen epithelium and the rumen bacteria involved in protein and energy metabolism promoted the utilization of dietary protein in calves fed OP.

## Introduction

In general, ruminants convert about 20% to 30% of dietary nitrogen (N) into animal protein, and the rest is excreted in urine and feces [1], which has adverse economic and environmental implications. It has become important to develop nutritional management practices to improve the nitrogen utilization efficiency (NUE) of ruminants.

In nature, several animals, microorganisms, and plants experience seasonal periods of nutrient enrichment followed by undernourishment. This nutritional oscillation seems to cause compensatory growth. Several studies have demonstrated that feeding with oscillating crude protein concentrations diet (OP) on a 48-h basis can enhance N retention of lambs [1, 2] and steers [3] relative to those consuming a similar amount of crude protein (CP) at a static concentration, reducing N release into the environment, but other studies have failed to demonstrate benefits of OP on N retention [4, 5]. When dietary CP has been oscillated, the growth and NUE of ruminants were at least unaffected or improved, which may be due to changes in absorption patterns of nitrogen compounds [2, 6]. The NUE of ruminants was affected by rumen fermentable energy [6]. Rumen microorganisms directly mediate the metabolism of dietary nutrients and affect feed efficiency, which is closely related to the rumen fermentation indexes. Metabolites in rumen fluid are also required for microbial growth [7], however, excessive or complex rumen microorganisms can lead to excessive decomposition of nutrients in the diet, resulting in wasted feed due to limited utilization capacity and a reduction in the animal’s growth rate [8]. Therefore, the effect of OP on N retention should be related to the rumen bacterial community and epithelial absorption patterns.

For the urea produced by the liver of ruminants, a proportion is excreted in urine and the balance is returned to gut through either blood transfer in epithelial tissue or via saliva [9]. A higher proportion of urea-N recycling into the rumen means a more efficient use of urea-N for microbial protein synthesis, which is a very efficient N salvaging mechanism for ruminants in a N deficiency period [10]. It has been suggested that the improved N retention by OP may be due to the increase of urea-N recycling [1, 2]. Most endogenously synthesized urea enters the rumen via blood and gut exchange of urea-N and NH_3_-N in rumen epithelium, which is affected by the concentrations of plasma urea-N and rumen NH_3_-N [10]. In addition, there are transporters in rumen epithelium that may be involved in the transport of plasma urea-N to the rumen [11, 12], but the results of different studies are inconsistent [1, 10]. Therefore, the role of urea-N recycling in improving NUE and its mechanism remain to be determined.

The purpose of this study was to investigate the effects and mechanism of OP on NUE of calves. It was hypothesized that OP might improve NUE, either by increasing urea-N recycling to rumen or changing the rumen bacteria community or epithelial absorption patterns during the low protein phase. An experiment was designed in which the diets were as similar as possible except for the CP level in the diets and the nutritional components of the diets fed by oscillating and stable feeding were consistent in a period of oscillation. The feed efficiency of calves was measured, the urea-N kinetics of calves evaluated by urea-^15^N^15^N labeling method, bacterial diversity and metabolites in rumen fluid was investigated by 16S rRNA sequencing and metabolomics and the differentially expressed genes in rumen epithelium and liver were evaluated by transcriptomics. The present experiment is the first to assess the physiological adaptive mechanisms for this dietary intervention by combining isotope labeling and multi-omic methods.

## Materials and methods

### Animals, design, and diets

Twelve 4-month-old Holstein male calves with a mean weight of 94.3 ± 6.5 kg, were housed in individual hutches at Henan Agricultural University, Practical and Teaching Base (Xuchang, China). Calves were randomly assigned into two dietary treatments (n = 6): 1) continuous feeding of a 149 g/kg CP diet (static protein diet, SP), 2) oscillating feeding of 125 and 173 g/kg CP diets at 2-d intervals (oscillating crude protein concentrations diet, OP). The ingredients and nutrient level of the basal diets are shown in Table 1. Calves were fed twice daily at 0800 h and 1700 h with equal amount of dry matter intake in the two groups.

**Table 1:** Ingredients and nutrient composition of diets.

Items	OP		SP
	High CP	Low CP	
**Ingredient, g/kg of dry matter**			
**Corn, ground**	337	420	378.5
**Soybean meal**	195	126	160.5
**Corn protein powder**	21.6	0	10.8
**Wheat bran**	63	71.5	67.25
**Soybean oil**	2.4	0	1.2
**Limestone**	12	11	11.5
**Dicalcium phosphate**	6	8.5	7.25
**Salt**	6.5	6.5	6.5
**Vitamin-mineral premix** ^**1**^	6.5	6.5	6.5
**Oat grass, chopped**	350	350	350
**Total**	1000	1000	1000
**Chemical composition, g/kg of dry matter (unless noted)**			
**Dry matter (g/kg of air dry matter)**	898	900	899
**Crude protein**	173	125	149
**Crude fat**	30.7	44.5	37.6
**Neutral detergent fiber**	342	344	343
**Acid detergent fiber**	117	120	118.5
**Crude ash**	47.2	44.3	45.75
**Calcium**	8.7	8.7	8.7
**Phosphorus**	4.8	4.8	4.8
**Net energy for maintenance (Mcal/kg)** ^**2**^	1.89	1.9	1.9
**Net energy for gain (Mcal/kg)** ^**2**^	1.25	1.25	1.25
**Gross Energy (Mcal/kg)**	4.38	4.33	4.36

OP, oscillating crude protein concentrations diet; SP, static protein diet.

^1^Containing per kg of supplement: 15,000 IU of vitamin A, 5,000 IU of vitamin D, 50 mg of vitamin E, 90 mg of Fe, 12.5 mg of Cu, 60 mg of Mn, 100 mg of Zn, 0.3 mg of Se, 1.0 mg of I, and 0.5 mg of Co.

^2^Calculated from tables of feed composition and nutritive values in China (2018 28^th^ Edition; database, 2018).

**Fig 1:**
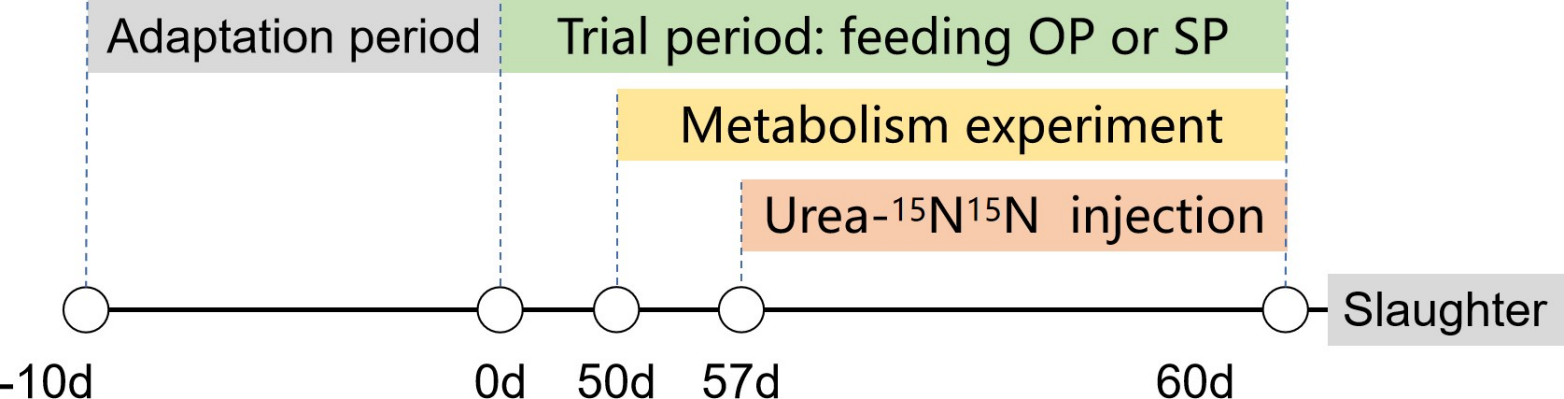
Diagram of experimental design. OP, oscillating crude protein concentrations diet; SP, static protein diet.

Experimental design is illustrated in Fig 1. The trial lasted for 60 days after 10 d adaptation. On day 50 of the trial period, each calf was placed in a specially designed metabolic cage. After 3 days of adaptation, the daily feed intake, refusals, urine, and fecal were weighed and sampled from day 53 to day 60 to examine the nutrient composition. Three calves with similar body weight were selected from each group to determine the urea-N kinetics using urea-^15^N^15^N isotope labeling. A saline solution (0.15 M NaCl) was infused continuously from the time the catheter was placed in the jugular vein of calves on the evening of day 57, until 0800 h on day 58 when the urea-^15^N^15^N as 99.16 atom % ^15^N (Shanghai Chemical Research Institute, Shanghai, China) was dissolved in sterile 0.15 M NaCl and then continuously injected for 48 hours. The urine and fecal samples were collected to examine the ^15^N enrichments. Urine and feces on day 57 were collected as ^15^N “background” enrichments.

The Theoretic Committee on Animal Protection and Use at Henan Agricultural University specifically approved this study, and all procedures involving calves were in compliance with the guidelines of the “Protocol of Animal Welfare for Bovine During Breeding, Transport, and Slaughter” (SN-T 3774–2014).

### Slaughtering procedure and tissue collection

Blood samples was obtained from the jugular vein after being fasted for 8h and centrifuged for the detection of Urea-N. Calves were slaughtered at the end of the trial period. The slaughtering and carcass-segmentation process conformed to the “Evaluating rule on the safety food quality of the slaughtered beef products” (SB/T 10364–2012). The rumen, reticulum, omasum, and abomasum were weighed without chyme. The rumen fluid was collected to measure pH, and the concentration of NH_3_-N and Volatile Fatty Acids (VFAs). Rumen fluid from four calves in each group with similar final body weight was collected for microbial DNA and metabolites extraction. The rumen epithelium and liver of animals infused with urea-^15^N^15^N were collected for total RNA extraction.

### Nutrient composition

The components of dry matter, CP, crude fat, neutral detergent fiber, acid detergent fiber, crude ash, calcium and phosphorus in feed, fecal and urine were measured according to the guidelines of National or Agricultural Industry Standards of the PRC, and the gross energy was measured using a microcomputer automatic calorimeter.

### Urea-N kinetics

The ^15^N enrichment in feces was analyzed at the Institute of Subtropical Agriculture, Chinese Academy of Sciences (Changsha, China) using stable isotope mass spectrometry. According to the method described by Wickersham et al. [13], ammonia was removed from urine using a cation exchange resin. Samples were then used to prepare ^28^N_2_, ^29^N_2_, and ^30^N_2_ gases using Hofmann degradation. The enrichment of these gas samples was analyzed at the Shanghai Chemical Research Institute (Shanghai, China) using an Agilent 7890B-7000C GC-MS instrument. The urea-N kinetics were estimated with the formula reported by Lobley et al. [9].

### Urea-N, NH_3_-N and VFAs

The urea-N concentration in plasma and urine were determined by diacetyl oxime colorimetry using a urea assay kit. The pH value of the rumen fluid was measured immediately and the samples were filtered through sterile gauze, 10 mL of which was mixed with 10% (*v/v*) one mL sulfuric acid and centrifuged (at 4300 × *g* for 20 min and then 12,000 × *g* for 20 min, 4°C) [14]. The supernatant of 1 mL was used to determine the concentration of NH_3_-N according to the method described by Broderick and Kang [15]. Another 1 mL of this supernatant was filtered through a 0.22-micron membrane to determine the VFAs with ion chromatography. Samples were injected with an autosampler into a Dionex AS11-HC column on a Sykam ion chromatography system. Chromatographic separation was carried out using a solvent system consisting of two eluents, a 0.5 mmol/L NaOH held for 25 min, then 50 mmol/L NaOH for 5 min and finally 0.5 mmol/L NaOH for 10 min, with all samples in duplicate.

### Transcriptome sequencing and analysis of differentially expressed genes (DEGs)

Total RNA was extracted using a mirVana miRNA Isolation Kit (Ambion-1561) following the manufacturer’s protocol. The libraries were sequenced on the Illumina HiSeqTM 2500 system and 125 bp paired-end reads were generated at Shanghai OE Biotech (Shanghai, China). These sequence data sets are publicly available through NCBI’s Sequence Read Archive, under accession number PRJNA661616 for rumen epithelium and PRJNA661470 for liver. Reads were mapped to the reference genome using HISAT2 [16]. The DEGs were identified using the DESeq. The values of *P* < 0.05 and FC > 2 or < 0.5 were set as the thresholds for significantly differential expressions. The Gene Ontology (GO) enrichment and Kyoto Encyclopedia of Genes and Genomes (KEGG) pathway enrichment analysis of the DEGs were performed using DAVID Functional Annotation Bioinformatics Microarray Analysis (https://david.ncifcrf.gov/).

Quantitative PCR (qPCR) was performed to validate the identified DEGs from the RNA-seq. The primers of *b-actin* (housekeeping gene) [17] and ten targeted genes, including five DEGs in the rumen epithelium and five DEGs in the liver were designed using BLAST (https://blast.ncbi.nlm.nih.gov/Blast.cgi) according to the available sequences in the NCBI (S1 Table). All samples were run in triplicate and the data were analyzed according to the 2^−ΔΔCT^ method [18].

### DNA extraction and 16S rRNA sequencing

Total DNA was extracted from the rumen fluid using a Bacterial DNA Kit (Omega). The V3 and V4 regions of the 16S rRNA gene were amplified by PCR using the universal primers 338F (5′-ACTCCTACGGGAGGCAGCAG-3′) and 806R (5′-GGACTACHVGGGTWTCTAAT-3′) [19]. All amplified libraries were sequenced using an Illumina HiSeq 2500 (Illumina, San Diego, CA) at Biomarker Technologies Co, Ltd. (Beijing, P.R. China). These sequence data sets are publicly available through NCBI’s Sequence Read Archive, under accession number PRJNA661397. The operational taxonomic units (OTUs) were annotated based on the Silva (bacteria) taxonomic database (Release 128, http://www.arb-silva.de) to generate species abundance tables at different taxonomic levels. Alpha-diversity was evaluated using Mothur software (version 1.30) [20]. Beta-diversity was analyzed using QIIME software (version 1.8.0) [21] and the distance between the samples was calculated by weighted UniFrac [22]. The use of LEfSe [23] was to determine the discriminative bacteria that most likely explain differences between calves fed OP and SP. This study used KEGG to annotate the differential functional genes of bacterial communities.

### Metabolomics analysis

Metabolites in 100 μL rumen fluid were extracted with 300 μL of methanol. Then, the supernatant was taken for the LC-MS/MS analyses using an UHPLC system (1290, Agilent Technologies) with a UPLC BEH Amide column (1.7 μm 2.1 × 100 mm, Waters) and coupled to TripleTOF 5600 (Q-TOF, AB Sciex) at Biomarker Technologies Co, Ltd. (Beijing, China). The ESI source conditions were set as follows: ion source gas 1 as 60 Psi, ion source gas 2 as 60 Psi, curtain gas as 35 Psi, source temperature of 650°C, and ion spray voltage floating (ISVF) 5000 V or -4000 V in positive or negative modes, respectively. An in-house MS2 database was used for metabolite identification. Differential metabolites were screened by combining the fold change (FC > 2), *P* value of the Student’s *t*-test (*P* < 0.05) and the VIP value of the OPLS-DA model (VIP > 1).

### Data processing and statistical analysis

Pearson’s correlation analysis between bacteria and fermentation index, and discriminative bacteria and differential metabolites in rumen was carried out and the thermogram was drawn using BMKCloud (www.biocloud.net). The data of stomachus compositus weights, rumen fermentation variables, nutrient utilization, urea-N kinetics, and relative gene expression were analyzed with unpaired Student’s *t*-tests using SPSS Statistics 22 (IBM., Armonk, NY, USA) to determine the effects of diets. The significance threshold for all statistical analyses was set to *P* < 0.05, and a tendency was declared at 0.05 ≤ *P* < 0.10.

## Results

### Stomachus compositus weights and rumen fermentation index

As shown in Table 2, the weights of total stomach (*P* = 0.069), rumen (*P* = 0.097) and omasum (*P* = 0.096) trend to be higher in calves fed OP compared with those fed SP. The NH_3_-N concentration in rumen fluid of calves fed OP was higher (*P* < 0.05) than those fed SP.

**Table 2:** Stomachus compositus weights and rumen fermentation index in calves fed OP and SP.

Items	OP	SP	SEM	*P-*value
**Stomachus compositus weights**				
**Total stomach (kg)**	5.3	4.8	0.25	0.069
**Rumen (kg)**	3.0	2.7	0.15	0.097
**Reticulum (kg)**	0.4	0.4	0.02	0.184
**Omasum (kg)**	1.1	0.9	0.10	0.096
**Abomasum (kg)**	0.8	0.7	0.06	0.274
**Rumen fermentation index**				
**pH**	5.85	5.85	0.05	0.971
**NH**_**3**_ **-N (mg/L)**	102.9	89.5	5.36	0.032
**Acetic acid (mg/L)**	1329.7	1341.5	270.34	0.966
**Propionic acid (mg/L)**	291.1	339.4	78.03	0.550
**Butyric acid (mg/L)**	141.4	137.4	24.59	0.874
**Acetic acid / Propionic acid (A/P)**	4.7	4.1	0.62	0.413
**Plasma urea-N (mmol/L)**	7.4	6.7	0.88	0.414

OP, oscillating crude protein concentrations diet; SP, static protein diet; SEM, standard error of mean.

### OP promotes the NUE and energy metabolism of calves

As shown in Table 3, the apparent crude fat digestibility was higher in calves fed OP than those fed SP (*P* < 0.05). The apparent digestibility of dry matter, neutral detergent fibers and acid detergent fiber were not significantly affected by diets (*P* > 0.05). For N metabolism, N intake, retained N, digestible N, apparent digestibility of N, and apparent utilization of N in calves fed OP were higher than those fed SP (*P* < 0.05). Fecal N, urine N, total excreted N, and fecal N/total excreted N were not influenced by diet (*P* > 0.05). The total excreted N/N intake in calves fed OP was lower than those fed SP (*P* < 0.05). In terms of energy metabolism, GE intake, digestive energy, net energy for maintenance, and combined net energy were higher in calves fed OP than those fed SP (*P* < 0.05), while the other indexes considered were not significant affected by diet (*P* > 0.05). In brief, OP promoted the NUE and energy metabolism of calves. Therefore, we speculate that OP affects the mechanism of nitrogen metabolism.

**Table 3:** Evolution of apparent nutrient digestibility, nitrogen metabolism and energy metabolism in calves fed OP and SP.

Items	OP	SP	SEM	*P-*value
**Apparent nutrient digestibility (%)**				
**Dry matter**	61.3	60.0	3.07	0.663
**Crude fat**	61.10	48.8	5.31	0.043
**Neutral detergent fibers**	43.0	40.3	2.03	0.212
**Acid detergent fiber**	38.4	35.3	3.38	0.382
**Nitrogen metabolism**				
**N intake (g/d)**	110.4	93.2	1.75	<0.01
**Fecal N (g/d)**	36.7	39.8	2.54	0.271
**Urine N (g/d)**	34.1	34.3	5.01	0.974
**Retained N (g/d)**	39.6	19.1	4.95	0.002
**Digestible N (g/d)**	73.7	53.4	2.57	<0.01
**Total excreted N (g/d)**	73.4	71.4	4.40	0.661
**Apparent digestibility of N (%)**	66.7	57.3	2.17	0.001
**Apparent utilization of N (%)**	35.9	20.4	4.80	0.009
**Total excreted N/N intake (%)**	66.4	76.7	3.88	0.024
**Fecal N/Total excreted N (%)**	46.2	47.9	5.93	0.784
**Energy metabolism**				
**Gross energy intake (MJ/d)**	83.7	80.0	1.54	0.034
**Fecal energy (MJ/d)**	28.1	29.1	1.20	0.428
**Urinary energy (MJ/d)**	3.5	2.8	0.57	0.230
**Methane energy (MJ/d)**	0.08	0.08	0.001	0.122
**Digestive energy (MJ/d)**	55.7	51.0	2.01	0.039
**Metabolic energy (MJ/d)**	52.1	48.1	2.01	0.072
**Net energy for maintenance (MJ/d)**	32.5	29.5	1.31	0.043
**Net energy for gain (MJ/d)**	19.8	17.4	1.10	0.056
**Combined net energy (MJ/d)**	26.7	23.9	1.27	0.049
**Metabolic rate of gross energy (%)**	62.0	59.9	1.75	0.256
**Metabolic rate of digestive energy (%)**	93.5	94.3	1.03	0.440
**Apparent digestibility of gross energy (%)**	65.7	63.4	2.49	0.378

OP, oscillating crude protein concentrations diet; SP, static protein diet; SEM, standard error of mean.

### Differences of urea-N kinetics between calves fed OP and SP

To explore the mechanism by which OP promotes the NUE, we first analyzed the urea-N kinetics of calves by urea-^15^N^15^N isotope labeling method during the low protein phase. As shown in Table 4, the urea-N production (UER, *P* = 0.051) and that entry to gastrointestinal (GER, *P* = 0.084) tended to increase, and urea-N reused for anabolism (UUA, *P* < 0.05) increased significantly in calves fed OP during the low protein phase compared with those fed SP. These data indicate that urea-N recycling contributed to improving NUE when dietary protein concentration was low. The other indexes were not affected by diets (*P* > 0.05).

**Table 4:** Evolution of urea-N kinetics in calves fed SP and OP.

Item	OP	SP	SEM	*P-*value
**Urea kinetic variables (g urea N/d)**				
**Urea synthesized (UER)**	83.6	80.1	0.98	0.051
**Eliminated in urine (UUE)**	29.5	30.2	0.41	0.429
**Entry to GIT (GER)**	54.1	49.8	1.27	0.084
**Return to ornithine cycle (ROC)**	28.1	27.2	0.92	0.656
**Excreted in feces (UFE)**	0.5	0.5	0.03	0.537
**Re-use for anabolism (UUA)**	25.4	22.2	0.82	0.017
**Fractional transfers**				
**Production to urine (UUE: UER)**	0.4	0.4	0.01	0.157
**Production to GIT (GER: UER)**	0.6	0.6	0.01	0.157
**GIT to ornithine cycle (ROC: GER)**	0.5	0.5	0.01	0.366
**GIT to feces (UFE: GER)**	0.01	0.01	0.001	0.983
**GIT to anabolism (UUA: GER)**	0.5	0.4	0.01	0.341

OP, oscillating crude protein concentrations diet; SP, static protein diet; SEM, standard error of mean; UER, urea-N entry rate; UUE, urinary urea-N elimination; GER, gastrointestinal entry rate; ROC, return to ornithine cycle; UFE, urea-N to fecal excretion; UUA, urea-N utilized for anabolism; GIT, gastrointestinal tract.

### DEGs in rumen epithelium and liver between calves fed OP and SP

**Fig 2:**
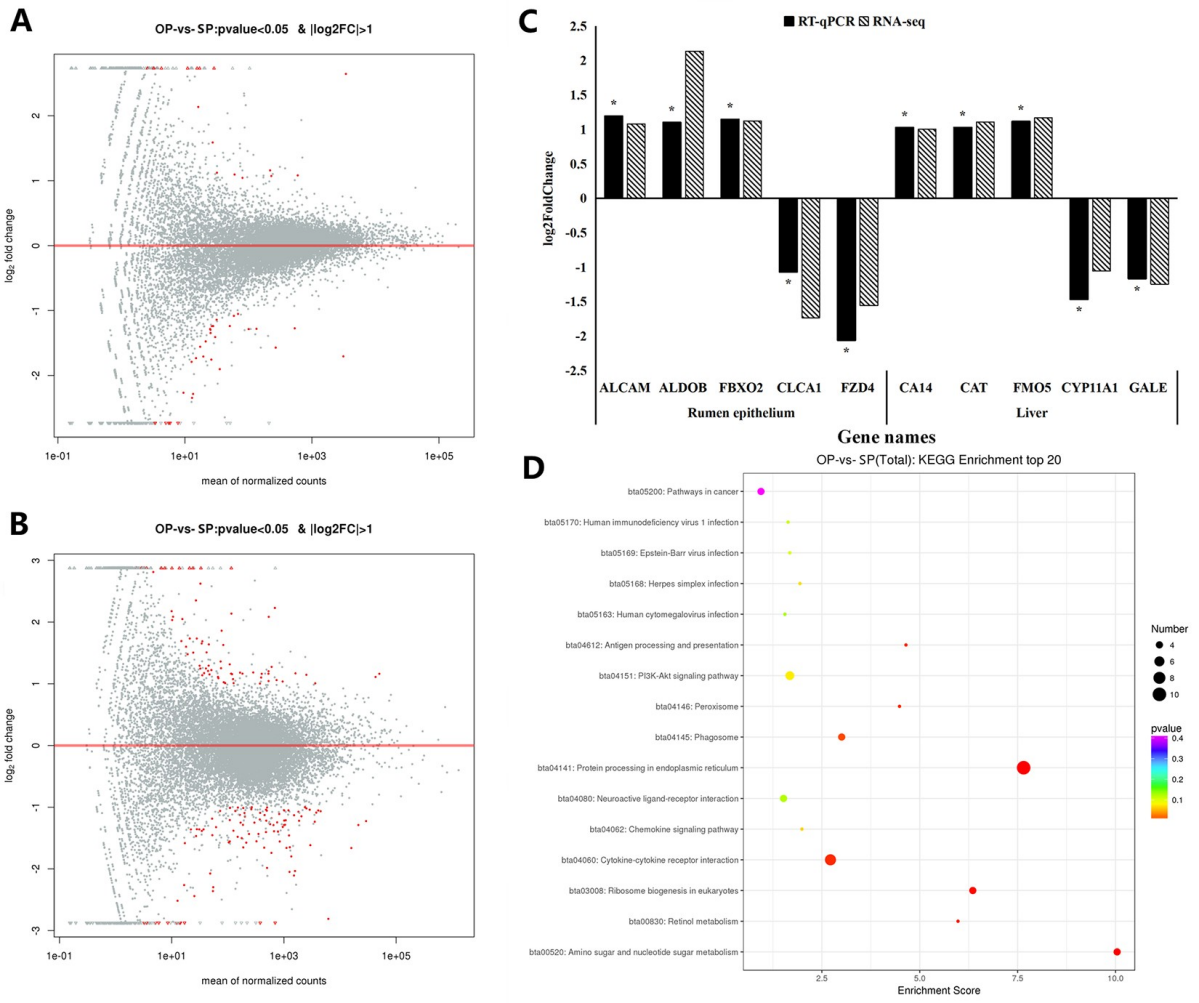
Analysis of differentially expressed genes (DEGs) in rumen epithelium and liver between calves fed OP and SP. DEGs in rumen epithelium (A) and liver (B), with red dots represent DEGs and gray dots represent all detected genes; (C) Fold-change of 10 genes determined by both RT-qPCR and RNA-sequencing methods; (D) KEGG annotate the DEGs in liver. OP, oscillating crude protein concentrations diet; SP, static protein diet.

We further analyzed gene expression in rumen epithelium and liver by transcriptome sequencing. There were 48 DEGs in the rumen epithelium, of which 18 were upregulated and 30 were downregulated (Fig 2A) in calves fed OP compared with those fed SP. There were 179 DEGs in the liver, of which 74 were upregulated and 105 were downregulated in calves fed OP compared with those fed SP (Fig 2B). The validation of ten DEGs, five in rumen epithelium and five in liver, by qPCR confirmed the accuracy of DESeq2 analysis results (Fig 2C).

We annotated the function of these DEGs on GO and KEGG. The DEGs in the rumen epithelium were significantly enriched in 10 GO terms, which included one GO term in the biological process (BP) category, seven GO terms in the cellular component (CC) category and two GO terms in the molecular function (MF) category (Table 5). Importantly, several DEGs including *C1RL*, *PRSS2*, and *TLL1* were associated with serine-type endopeptidase activity and *CLSTN3*, *PRSS2*, and *TLL1* with MF calcium ion binding, which may be involved in the process of nitrogen metabolism.

**Table 5:** Gene ontology analysis of differentially expressed genes in rumen epithelial of calves fed OP and SP.

Category	Gene ontology category (Accession No.)	Observed genes	Gene number	Enrichment score	*P-*value
**Biological process**	cell adhesion (GO:0007155)	ALCAM; FREM2; HEPACAM	3	4.130	0.006
**Cellular component**	cell-cell junction (GO:0005911)	FZD4; HEPACAM; LOC618633	3	10.841	0.000
	microtubule organizing center (GO:0005815)	ALDOB; PIK3R5; RPP25	3	8.953	0.000
	extracellular exosome (GO:0070062)	C1RL; FREM2; RIMS2; SERPINI2; SHROOM2	5	3.362	0.003
	extracellular space (GO:0005615)	C1RL; CLCA1; COL24A1; PRSS2; SERPINI2; ULBP27	6	2.498	0.009
	cell surface (GO:0009986)	CLSTN3; FZD4; ULBP27	3	3.368	0.012
	integral component of plasma membrane (GO:0005887)	ALCAM; CLCA1; FZD4; KCNH8; LOC100337457	5	2.455	0.015
	extracellular region (GO:0005576)	COL24A1; LOC101905630; PRSS2; TLL1; ULBP27	5	2.112	0.029
**Molecular function**	serine-type endopeptidase activity (GO:0004252)	C1RL; PRSS2; TLL1	3	8.673	0.000
	calcium ion binding (GO:0005509)	CLSTN3; PRSS2; TLL1	3	2.313	0.040

Gene ontology categories with corrected *P* values of enrichment significance below 0.05 are shown. OP, oscillating crude protein concentrations diet; SP, static protein diet.

The DEGs in the liver were significantly enriched 27 GO terms, including eight in BP, ten in CC, and nine in MF (S2 Table). Eight KEGG pathways (Fig 2D), including protein processing in endoplasmic reticulum, amino sugar and nucleotide sugar metabolism, ribosome biogenesis in eukaryotes, and retinol metabolism, were enriched, indicating that OP significantly alter the process of liver metabolism in calves.

### Bacterial community characteristics and differences between calves fed OP and SP

**Fig 3:**
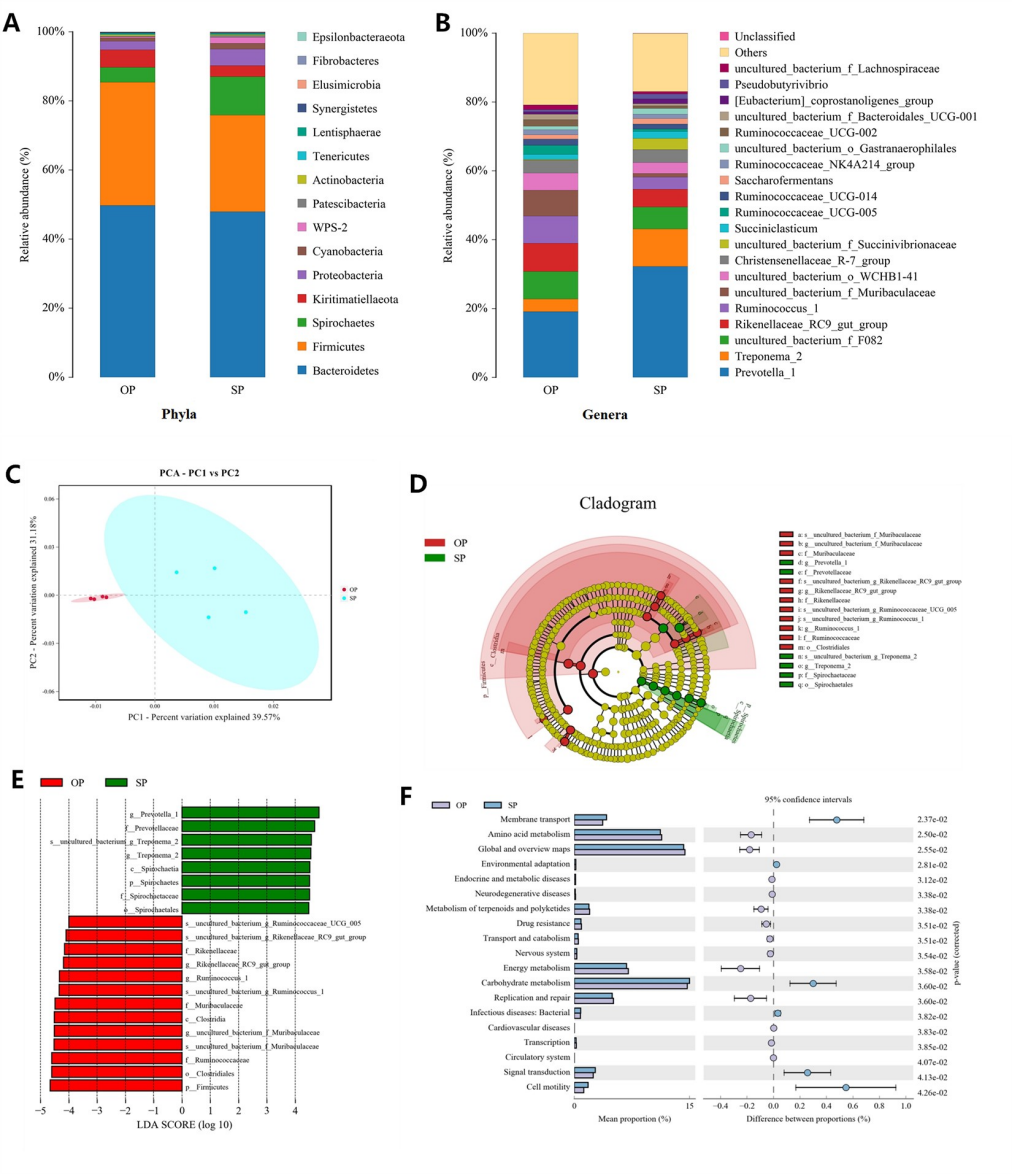
16S rRNA genes sequencing analysis of bacterial diversity in rumen fluid. (A) Phyla- and (B) genera-level composition of the rumen bacteria (top 20); (C) PCA analyses of beta diversity; (D) LEfSe analysis indicated the biomarker bacteria and (E) Histograms of linear discriminant analysis (LDA) for the differential biomarker; (F) KEGG annotates the differential functional genes of bacterial communities. OP, oscillating crude protein concentrations diet; SP, static protein diet.

To confirm whether rumen bacterial play a role in improving NUE, we evaluated the rumen bacterial community characteristics and differences between the two groups by 16S rRNA sequencing. As shown in Fig 3A, the bacterial community was consisted of 15 phyla, of which *Bacteroidetes*, *Firmicutes* and *Spirochaetes* were the three most abundant phyla. The dominant genera were *Prevotella*_1, followed by *Treponema*_2, and uncultured_bacterium_f_*F082* (Fig 3B).

Alpha diversity estimators of community are shown in Table 6. According to the Ace and Chao indexes, the bacterial richness was no significant difference between the two groups (*P* > 0.05). The Shannon and Simpson indexes showed that the bacterial community diversity in rumen of calves fed OP was higher (*P* < 0.05) than those fed SP. The beta diversity analysis showed two different clusters, indicating that the bacteria community structure and composition were significantly different between the two group (Fig 3C).

**Table 6:** OTU richness and alpha-diversity indices of rumen bacteria in calves fed SP and OP.

Items	OP	SP	SEM	*P-*value
**chao** **1**	638.28	653.37	6.93	0.311
**Ace**	621	638	5.62	0.130
**Shannon**	4.93	4.61	0.07	0.004
**Simpson**	0.016	0.031	0.003	0.001
**Observed OTUs**	680	720	5.07	
**Coverage**	>99%	>99%	0.000	

OP, oscillating crude protein concentrations diet; SP, static protein diet; SEM, standard error of mean.

The LEfSe analysis revealed 12 discriminative features (LDA score > 4) from phylum to genus between calves fed OP and SP (Fig 3D and 3E). The phylum *Firmicutes* were more abundant in calves fed OP, while *Spirochaetes* were more abundant in calves fed SP. At the genera level, uncultured_bacterium_f_*Muribaculaceae*, *Rikenellaceae_RC9_*gut*_*group and *Ruminococcus*_1 was more abundant in calves fed OP, while *Prevotella*_1 *and Treponema*_2 was more abundant in calves fed SP.

OP altered the microbiota-associated functions in calves. The different functional bacteria were enriched in 19 KEGG metabolic pathways (Fig 3F). The differential metabolic pathways with high relative abundance include membrane transport, amino acid metabolism, global and overview maps, metabolism of terpenoids and polyketides, energy metabolism, carbohydrate metabolism, replication and repair, signal transduction and cell motility. Therefore, the different bacteria may change the composition of rumen metabolites.

### OP changed the metabolites in rumen fluid of calves

Next, we identified the metabolites in rumen fluid via untargeted metabolomic analyses. As shown in Table 7, only 15 different metabolites affected by diets could be identified using the currently available metabolite databases, of which six metabolites was downregulated and nine metabolites were upregulated in calves fed OP compared to those fed SP. These differential metabolites are related to the composition of bacterial community, just as rumen fermentation indexes.

**Table 7:** Differential metabolites in the rumen of calves fed OP and SP.

Metabolites	mzmed	rtmed	log2FC	*P* value	VIP	Regulated
**Negative**						
**Maleamic acid**	114.02	21.45	2.29	0.00	2.21	up
**Adenine**	134.05	149.18	1.41	0.03	1.83	up
**L-Erythro-4-Hydroxyglutamic acid**	144.03	22.01	1.93	0.01	2.12	up
**N-Acetyl-L-aspartic acid**	174.04	361.71	1.46	0.02	2.03	up
**L-Tryptophan**	203.08	282.09	1.65	0.04	1.92	up
**Adenosine 3’,5’-cyclic phosphate (cAMP)**	328.05	245.59	1.31	0.04	1.88	up
**Vindoline**	437.22	351.69	-1.90	0.00	2.13	down
**Sildenafil**	473.19	288.05	-2.82	0.00	2.36	down
**Positive**						
**4-Aminophenol**	109.05	149.66	1.05	0.02	1.91	up
**Adenine**	136.06	149.58	1.24	0.02	1.95	up
**Kojic Acid**	160.06	19.54	1.19	0.03	2.07	up
**Phenylacetylglycine**	235.11	84.61	-1.03	0.01	2.10	down
**Terpineol**	347.24	208.14	-1.07	0.04	1.86	down
**L-Tetrahydropalmatine**	419.20	220.36	-1.61	0.02	1.94	down
**Diethyltoluamide**	421.22	343.77	-1.79	0.02	1.91	down

OP, oscillating crude protein concentrations diet; SP, static protein diet; mzmed, mass-to-charge ratio of metabolites; rtmed, retention time of metabolites; FC, fold change; VIP, variable importance in the projection. Regulated “Up” represent a higher abundance in calves fed OP and “down” represent a higher abundance in calves fed SP.

### Relationship between bacteria and fermentation indexes or metabolites in rumen

**Fig 4:**
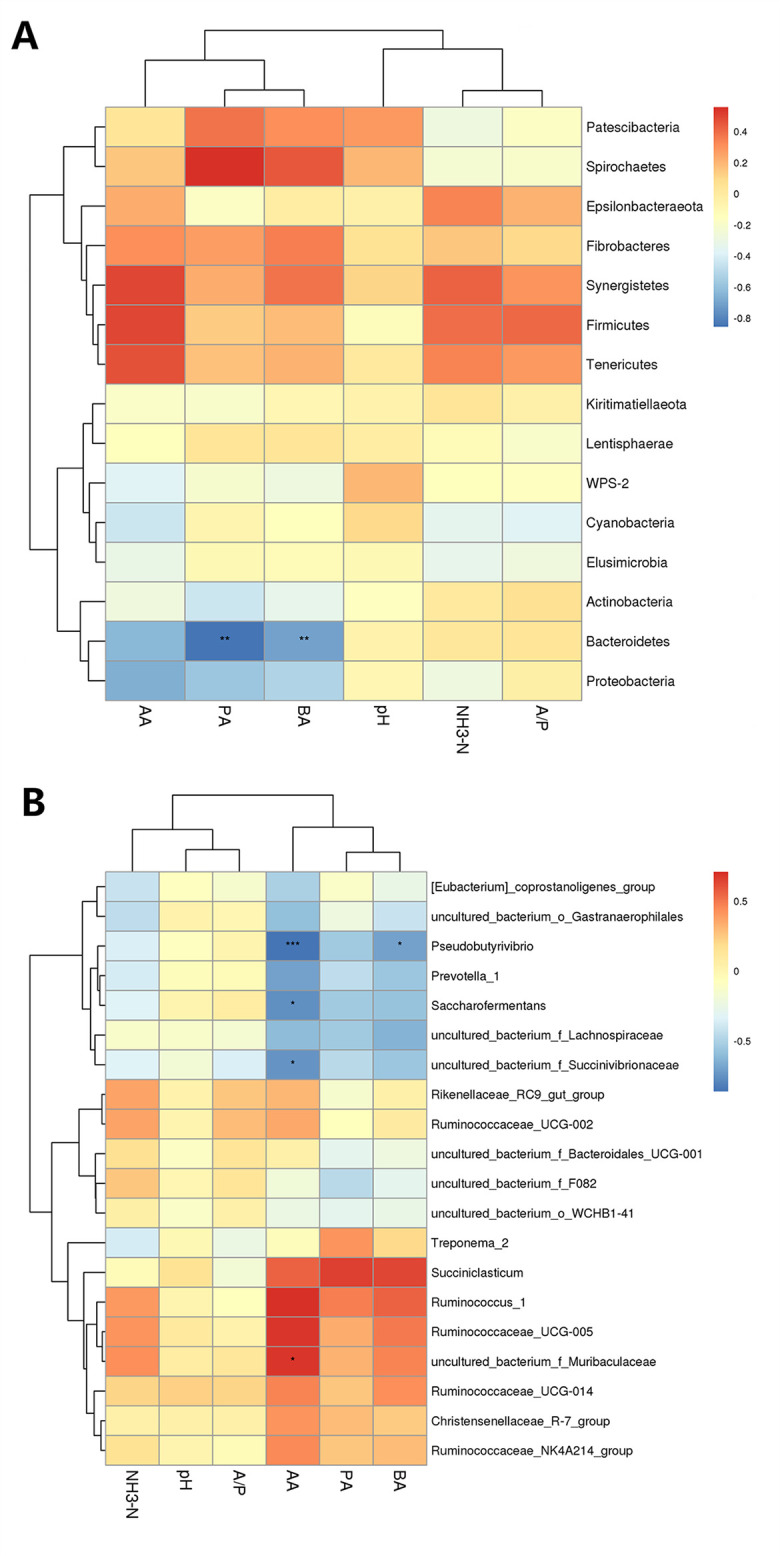
Thermogram of Pearson’s correlation between bacterial abundance (at the (A) phyla- and (B) genera level) and fermentation index. The fermentation indexex included pH, acetic acid/propionic acid (A/P), and the concentrations of NH_3_-N, acetic acid (AA), propionic acid (PA) and butyric acid (BA).

**Fig 5:**
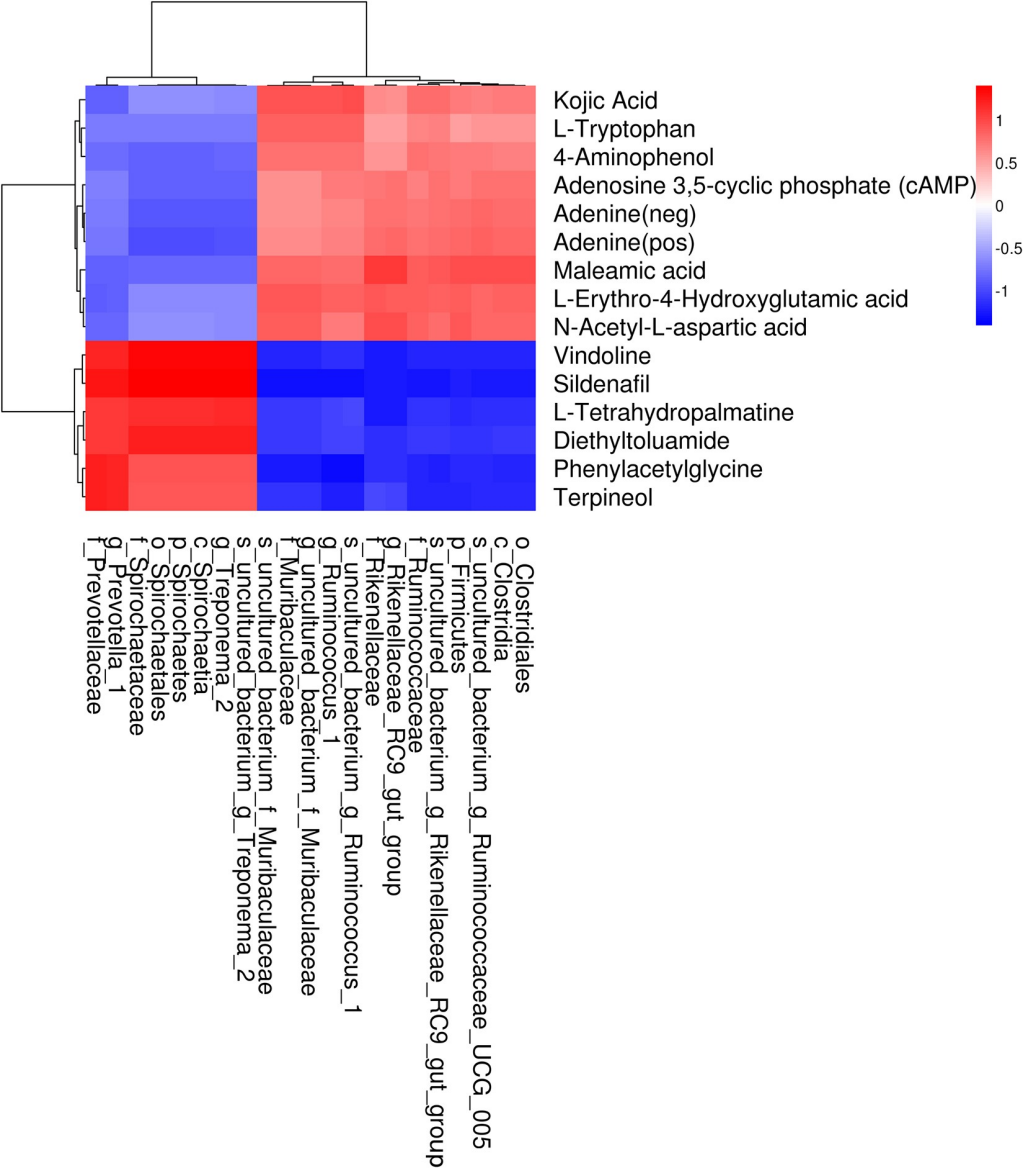
Thermogram of Pearson’s correlation between discriminative bacteria and differential metabolites in rumen.

Therefore, we analyzed the Pearson’s correlation between bacteria and fermentation index, and discriminative bacteria and differential metabolites in rumen. The main fermentation indexes related to bacteria in the rumen were the concentrations of acetic acid, propionic acid and butyric acid (Fig 4A and 4B). Among them, *Bacteroidetes* was negatively correlated with propionic acid and butyric acid in the rumen of calves (Fig 4A). The genus uncultured_bacterium_f_*Muribaculaceae* was positively correlated with acetic acid (Fig 4B). As shown in Fig 5, a total of 59 significant correlations were identified between the differential metabolites and discriminative bacteria in rumen (*P* < 0.01). At the genera level, *Prevotella*_1 was negatively correlated with kojic acid, maleamate, L-erythro-4-hydroxyglutamate, and N-acetyl-L-aspartate, and positively correlated with vindoline, sildenafil, phenylacetylglycine, and terpineol, suggesting that it may participate in the metabolism of some amino acids in rumen. In addition, the uncultured_bacterium_f_*Muribaculaceae* was positively correlated with kojic acid, L-erythro-4-hydroxyglutamate, and N-acetyl-L-aspartate, and *Ruminococcus*_1 was positively correlated with kojic acid, L-tryptophan, Maleamic acid, and L-Erythro-4-Hydroxyglutamic acid. The abundance of these two bacteria in calves fed OP was higher than those fed SP, so it may be involved in the metabolism of nitrogen compounds in rumen, providing absorbable amino acids to the host.

## Discussion

In the present study, OP promoted the N retention and NUE of calves, which agrees with the results of earlier studies on lambs [1, 2] and steers [3], indicating that the change of dietary CP concentrations in the present study may be synchronized with the retention time of diet in gut under the 48-h oscillating regimen [4]. This is great enough to reduce the amount of N emitted by ruminants to the environment for the same intake. In addition, more N-yielding substrates and larger total stomach weight require more energy degradation in rumen [5, 6]. The mass of gastrointestinal organs may ultimately affect energy utilization in ruminants [5]. When calves fed OP, the weight of total stomach, rumen and omasum tend to be higher, which was consistent with the higher apparent crude fat digestibility and energy metabolism.

Urea-N recycling provides available N for rumen microbial protein synthesis, which is more important when dietary N supply is deficient [10]. Urea is synthesized primarily in the liver [9], and its process is regulated by carbamoyl phosphate synthase 1, ornithine transcarbamylase, argininosuccinate synthetase, argininosuccinate lyase and arginase [24]. In this study, OP increased the UER of calves, while did not change the mRNA abundance of the five catalytic enzymes genes in liver during the low protein phase. Therefore, there may be other factors regulating urea synthesis in the liver. Energy metabolism affects urea-N recycling. Sufficient available carbohydrates in rumen promote the transfer of endogenous urea-N to gut rather than excretion in urine [25]. This study confirmed the above viewpoint. We found an increase in the gastrointestinal entry rate of urea during the low protein phase, which was consistent with the higher apparent crude fat digestibility and energy metabolism in calves fed OP. This also proved an adequate available energy in the experimental diets. The urea-N that was transferred to the gut is hydrolyzed by bacteria to release NH_3_-N, which is then used by microbial or mammalian metabolism [9]. Lambs had greater microbial non-NH_3_-N supply when fed oscillating compared with static dietary CP concentrations, which may be related to the incorporation of recycled urea-N [1]. In the present study, OP increased the GER that reused for anabolism during the low protein phase, which was consistent with the higher NH_3_-N concentration. In conclusion, the higher UER, GER and UUA were consistent with the higher NUE in calves fed OP, indicating that the improvement N retention could be attributed to urea-N transfer to gut and then reuse for anabolism when dietary protein concentration was low.

Rumen epithelium is responsible for the absorption and transportation of nutrients in the rumen. Several studies have reported that there are transporters in rumen epithelium that may be involved in the transport of plasma urea-N to the rumen, such as urea transporter B (*UT-B*), aquaglycoporin 3 (*bAQP3*) and G protein-coupled receptors (*GPR*) 4 and 41 [11, 12]. In this study, OP changed the amount of GER in calves, but did not change the expression of these transporter genes in rumen epithelium during the low protein phase. Therefore, the role of these transporters in the urea-N recycling remains to be determined, or the gene transcription level cannot represent protein abundance. According to transcriptome functional analysis, DEGs in rumen epithelium between calves fed OP and SP were significantly enriched 10 GO terms. The category of MF is based on serine-type endopeptidase activity, which catalyzes the hydrolysis of internal, alpha-peptide bonds in a polypeptide chain by a catalytic mechanism. The complement component *C1RL* (r-subcomponent like) is a serine protease that mediates the proteolytic cleavage of haptoglobin in the endoplasmic reticulum [26]. We speculated that the DGEs serine protease 2 (*PRSS2*) and *C1RL*, with higher expression in calves fed OP, may participate in the peptide metabolism in the rumen epithelium. Studies have shown that dietary N concentration of goats regulates intestinal Ca transport by Ca-transporting proteins [27]. The calcium ion, whose activity may be regulated by the Na^+^-K^+^ pump, participates in regulating lipid metabolism in adipose tissue [28]. Our results suggest that OP altered the molecular function of calcium ion binding in the rumen epithelium of calves. These results indicated that OP may induce calcium ion absorption or change the nutrient metabolism through calcium ion binding in rumen epithelium of calves. These data confirmed that OP promoted NUE partly by increasing the subsequent digestion and absorption of microbial protein or other nitrogenous compounds by the gastrointestinal tract [2] during the low protein phase.

Rumen and its microbiota play an important role in the degradation of feedstuffs. These microorganisms break down ingested compounds into their subcomponents, and provide nutrients to the host animal [29]. The effect of OP on rumen bacterial community diversity of calves during the low protein phase was analyzed by 16S rRNA sequencing. Consistent with other studies [30–32], *Bacteroidetes* and *Firmicutes* were the most abundant two phyla, and *Prevotella*_1 represented the highest percentage among the identified genera in the rumen of calves despite the diets. *Bacteroidetes* is the primary degraders of polysaccharides [25]. As the most abundant genus in the phylum *Bacteroidetes*, *Prevotella*_1 had positive effects on VFAs concentrations in the rumen of dairy cows [32]. However, we found no such relationship in calves. It has been reported that some members of the genus *Prevotella* participate in the degradation of oligopeptides into amino acids in rumen [30]. Similarly, in this study, correlation analysis revealed that *Prevotella*_1 may participate in the metabolism of some amino acids in rumen. *Prevotella*_1 was also positively correlated with NH_3_-N concentration in the rumen of dairy cows [32]. On the contrary, the concentration of NH_3_-N was increased while the abundance of *Prevotella*_1 decreased in the rumen liquid of calves fed OP, which was consistent with the study on Hu Lambs with dietary urea supplementation [33]. Also, the calves fed OP had higher NUE. Therefore, the abundance of *Prevotella_*1 could not be used as an accurate indicator of protein metabolism in rumen. Genomic data suggested that populations of *Muribaculaceae* are equipped with fermentation pathways to produce succinate, acetic acid and propionic acid by degrading plant glycans, host glycans and α-glucans [34]. In this study, uncultured_bacterium_f_*Muribaculaceae* was positively correlated with acetic acid, and *Muribaculaceae* was also correlated with propionic acid and butyric acid in gut [35, 36], which supported that it was multifunctional in carbohydrates degradation and could occupy different niches in the common community [37]. The bacteria within the family *Muribaculaceae* harbor a specific urease, which exhibits specificity in N utilization [34]. In this study, the abundance of uncultured_bacterium_f_*Muribaculaceae* was higher in calves fed OP, which may be involved in the metabolism of nitrogen compounds. Therefore, it is necessary to further understand the functions of this family in the rumen at the levels of genus, species, and/or strain. *Firmicutes* are major degraders of insoluble fiber [38]. Butyrate-producing *Ruminococcus*_1 belonging to this phylum may promote fiber degradation and were negatively correlated with propionic acid and isobutyrate concentrations in the rumen of dairy cows [31]. In theory, more vigorous carbohydrate metabolism is conducive to the absorption and utilization of nitrogen compounds by the host. The *Ruminococcus*_1 abundance in the rumen of calves fed OP was higher than those fed SP, which was consistent with NUE. These results support the view that *Ruminococcus*_1 in rumen can promote the utilization of dietary N by the host [39]. In summary, these bacteria involved in protein and energy metabolism promoted the utilization of dietary protein in the rumen of calves fed OP. Future research should focus on the dynamics of rumen bacterial flora and epithelial gene transcription patterns throughout the oscillation period, to better explore the mechanism of N retention.

## Conclusion

The change of dietary CP concentrations under the 48-h oscillating regimen may be an effective tool for producers to reduce the pollution of N emissions as it increased NUE of calves. Urea-N recycling contributed to improving NUE when dietary protein concentration was low. Furthermore, OP promoted NUE mainly by increasing the utilization of dietary protein and cycled urea-N by rumen microorganisms and the digestion and absorption of nitrogen compounds in rumen epithelium during the low protein phase. Unfortunately, these results have a limitation as all tissue and fluid samples were collected at slaughter during the low protein phase of the oscillation. The oscillation treatment is a dynamic metabolism in time, so further study should focus on the dynamics throughout the oscillation period.

## Supporting information

S1 Table Primers used for quantitative real-time PCR. (DOCX)

S2 Table Gene ontology analysis of differentially expressed genes in liver. Gene ontology categories with corrected *P* values of enrichment significance below 0.05 are shown.
(DOCX)

## References

[1] Doranalli K, Penner G, Mutsvangwa T. Feeding oscillating dietary crude protein concentrations increases nitrogen utilization in growing lambs and this response is partly attributable to increased urea transfer to the rumen. The Journal of nutrition. 2011;141(4):560–7. doi: 10.3945/jn.110.133876 . 21310865

[2] Archibeque S, Freetly H, Ferrell C. Net portal and hepatic flux of nutrients in growing wethers fed high-concentrate diets with oscillating protein concentrations. Journal of animal science. 2007;85(4):997–1005. doi: 10.2527/jas.2006-547 . 17145976

[3] Cole N, Greene L, McCollum F, Montgomery T, McBride K. Influence of oscillating dietary crude protein concentration on performance, acid-base balance, and nitrogen excretion of steers. Journal of animal science. 2003;81(11):2660–8. doi: 10.2527/2003.81112660x . 14601868

[4] Cole N. Nitrogen retention by lambs fed oscillating dietary protein concentrations. Journal of animal science. 1999;77(1):215–22. doi: 10.2527/1999.771215x . 10064047

[5] Ludden P, Wechter T, Hess B. Effects of oscillating dietary protein on nutrient digestibility, nitrogen metabolism, and gastrointestinal organ mass in sheep. Journal of animal science. 2002;80(11):3021–6. doi: 10.2527/2002.80113021x . 12462272

[6] Reynolds C, Kristensen N. Nitrogen recycling through the gut and the nitrogen economy of ruminants: an asynchronous symbiosis. Journal of animal science. 2008;86:E293–305. doi: 10.2527/jas.2007-0475 . 17940161

[7] Ogunade I, Schweickart H, Mccoun M, Cannon K, Mcmanus C. Integrating 16S rRNA Sequencing and LC–MS-Based Metabolomics to Evaluate the Effects of Live Yeast on Rumen Function in Beef Cattle. Animals. 2019;9(1). 10.3390/ani9010028 PMC6356510 30669471

[8] Li F, Hitch T, Chen Y, Creevey C, Guan L. Comparative metagenomic and metatranscriptomic analyses reveal the breed effect on the rumen microbiome and its associations with feed efficiency in beef cattle. Microbiome. 2019;7(1):6. doi: 10.1186/s40168-019-0618-5. 30642389 PMC6332916

[9] Lobley G, Bremner D, Zuur G. Effects of diet quality on urea fates in sheep as assessed by refined, non-invasive [15N15N]urea kinetics. The British journal of nutrition. 2000;84(4):459–68. doi: 10.1017/s0007114500001768 . 11103216

[10] Muscher A, Schröder B, Breves G, Huber K. Dietary nitrogen reduction enhances urea transport across goat rumen epithelium. Journal of animal science. 2010;88(10):3390–8. doi: 10.2527/jas.2010-2949 . 20581287

[11] Berends H, van den Borne J, Røjen B, van Baal J, Gerrits W. Urea recycling contributes to nitrogen retention in calves fed milk replacer and low-protein solid feed. The Journal of nutrition. 2014;144(7):1043–9. doi: 10.3945/jn.114.191353 . 24812069

[12] Lu Z, Gui H, Yao L, Yan L, Martens H, Aschenbach J, et al. Short-chain fatty acids and acidic pH upregulate UT-B, GPR41, and GPR4 in rumen epithelial cells of goats. American journal of physiology Regulatory, integrative comparative physiology. 2015;308(4):R283–93. doi: 10.1152/ajpregu.00323.2014 . 25519731

[13] Wickersham T, Titgemeyer E, Cochran R, Wickersham E, Moore E. Effect of frequency and amount of rumen-degradable intake protein supplementation on urea kinetics and microbial use of recycled urea in steers consuming low-quality forage. Journal of animal science. 2008;86(11):3089–99. doi: 10.2527/jas.2007-0326 . 18539827

[14] Saeedi S, Dayani O, Tahmasbi R, Khezri A. Effect of supplementation of calf starter with fennel powder on performance, weaning age and fermentation characteristics in Holstein dairy calves. Journal of animal physiology animal nutrition. 2017;101(1):81–7. doi: 10.1111/jpn.12511 . 28063208

[15] Broderick G, Kang J. Automated simultaneous determination of ammonia and total amino acids in ruminal fluid and in vitro media. Journal of dairy science. 1980;63(1):64–75. doi: 10.3168/jds.S0022-0302(80)82888-8 . 7372898

[16] Kim D, Langmead B, Salzberg S. HISAT: a fast spliced aligner with low memory requirements. Nature methods. 2015;12(4):357–60. doi: 10.1038/nmeth.3317 . 25751142 PMC4655817

[17] Steele M, Schiestel C, AlZahal O, Dionissopoulos L, Laarman A, Matthews J, et al. The periparturient period is associated with structural and transcriptomic adaptations of rumen papillae in dairy cattle. Journal of dairy science. 2015;98(4):2583–95. doi: 10.3168/jds.2014-8640 . 25682143

[18] Livak KJ. Analysis of relative gene expression data using real-time quantitative PCR and the 2(-Delta Delta C(T)) Method. Methods (San Diego, Calif). 2001;4(25). 10.1006/meth.2001.1262 11846609

[19] Mori H, Maruyama F, Kato H, Toyoda A, Dozono A, Ohtsubo Y, et al. Design and experimental application of a novel non-degenerate universal primer set that amplifies prokaryotic 16S rRNA genes with a low possibility to amplify eukaryotic rRNA genes. DNA research. 2014;21(2):217–27. doi: 10.1093/dnares/dst052 . 24277737 PMC3989492

[20] Schloss P, Westcott S, Ryabin T, Hall J, Hartmann M, Hollister E, et al. Introducing mothur: open-source, platform-independent, community-supported software for describing and comparing microbial communities. Applied environmental microbiology. 2009;75(23):7537–41. doi: 10.1128/AEM.01541-09 . 19801464 PMC2786419

[21] Caporaso J, Kuczynski J, Stombaugh J, Bittinger K, Bushman F, Costello E, et al. QIIME allows analysis of high-throughput community sequencing data. Nature methods. 2010;7(5):335–6. doi: 10.1038/nmeth.f.303 . 20383131 PMC3156573

[22] Lozupone C, Knight R. UniFrac: a new phylogenetic method for comparing microbial communities. Applied environmental microbiology. 2005;71(12):8228–35. doi: 10.1128/AEM.71.12.8228-8235.2005 . 16332807 PMC1317376

[23] Segata N, Izard J, Waldron L, Gevers D, Miropolsky L, Garrett W, et al. Metagenomic biomarker discovery and explanation. Genome biology. 2011;12(6):R60. doi: 10.1186/gb-2011-12-6-r60. 21702898 PMC3218848

[24] Moedas M, Adam A, Farelo M, IJlst L, Chamuleau R, Hoekstra R, et al. Advances in methods for characterization of hepatic urea cycle enzymatic activity in HepaRG cells using UPLC-MS/MS. Analytical biochemistry. 2017;535:47–55. doi: 10.1016/j.ab.2017.07.025 . 28757091

[25] Lapébie P, Lombard V, Drula E, Terrapon N, Henrissat B. Bacteroidetes use thousands of enzyme combinations to break down glycans. Nature communications. 2019;10(1):2043. doi: 10.1038/s41467-019-10068-5. 31053724 PMC6499787

[26] Shi L, Zhu B, Xu M, Wang X. Selection of AECOPD-specific immunomodulatory biomarkers by integrating genomics and proteomics with clinical informatics. Cell biology toxicology. 2018;34(2):109–23. doi: 10.1007/s10565-017-9405-x . 28779230

[27] Elfers K, Wilkens M, Breves G, Muscher-Banse A. Modulation of intestinal calcium and phosphate transport in young goats fed a nitrogen- and/or calcium-reduced diet. The British journal of nutrition. 2015;114(12):1949–64. doi: 10.1017/S000711451500375X . 26443238

[28] Cui H, Yang S, Zheng M, Liu R, Zhao G, Wen J. High-salt intake negatively regulates fat deposition in mouse. Scientific reports. 2017;7(1):2053. doi: 10.1038/s41598-017-01560-3. 28515432 PMC5435674

[29] Matthews C, Crispie F, Lewis E, Reid M, O’Toole P, Cotter PJGm. The rumen microbiome: a crucial consideration when optimising milk and meat production and nitrogen utilisation efficiency. 2019;10(2):115–32. doi: 10.1080/19490976.2018.1505176 . 30207838 PMC6546327

[30] Latham E, Weldon K, Wickersham T, Coverdale J, Pinchak W. Responses in the rumen microbiome of Bos taurus and indicus steers fed a low-quality rice straw diet and supplemented protein. Journal of animal science. 2018;96(3):1032–44. doi: 10.1093/jas/sky023 . 29617868 PMC6093561

[31] Pan X, Xue F, Nan X, Tang Z, Wang K, Beckers Y, et al. Illumina Sequencing Approach to Characterize Thiamine Metabolism Related Bacteria and the Impacts of Thiamine Supplementation on Ruminal Microbiota in Dairy Cows Fed High-Grain Diets. Frontiers in microbiology. 2017;8:1818. doi: 10.3389/fmicb.2017.01818. 28979254 PMC5611408

[32] Sun Z, Yu Z, Wang B. Perilla frutescens Leaf Alters the Rumen Microbial Community of Lactating Dairy Cows. Microorganisms. 2019;7(11). doi: 10.3390/microorganisms7110562. 31766265 PMC6921060

[33] Li Z, Mu C, Xu Y, Shen J, Zhu W. Changes in the Solid-, Liquid-, and Epithelium-Associated Bacterial Communities in the Rumen of Hu Lambs in Response to Dietary Urea Supplementation. Frontiers in microbiology. 2020;11:244. doi: 10.3389/fmicb.2020.00244. 32153533 PMC7046558

[34] Ormerod K, Wood D, Lachner N, Gellatly S, Daly J, Parsons J, et al. Genomic characterization of the uncultured Bacteroidales family S24-7 inhabiting the guts of homeothermic animals. Microbiome. 2016;4(1):36. doi: 10.1186/s40168-016-0181-2. 27388460 PMC4936053

[35] Evans CC, Lepard KJ, Kwak JW, Stancukas MC, Laskowski S, Dougherty J, et al. Exercise Prevents Weight Gain and Alters the Gut Microbiota in a Mouse Model of High Fat Diet-Induced Obesity. Plos One. 2014;9(3):e92193. doi: 10.1371/journal.pone.0092193 24670791 PMC3966766

[36] Smith B, Miller R, Ericsson A, Harrison D, Strong R, Schmidt T. Changes in the gut microbiome and fermentation products concurrent with enhanced longevity in acarbose-treated mice. BMC microbiology. 2019;19(1):130. doi: 10.1186/s12866-019-1494-7. 31195972 PMC6567620

[37] Lagkouvardos I, Lesker T, Hitch T, Gálvez E, Smit N, Neuhaus K, et al. Sequence and cultivation study of Muribaculaceae reveals novel species, host preference, and functional potential of this yet undescribed family. Microbiome. 2019;7(1):28. doi: 10.1186/s40168-019-0637-2. 30782206 PMC6381624

[38] Berry D. The emerging view of Firmicutes as key fibre degraders in the human gut. Environmental microbiology. 2016;18(7):2081–3. doi: 10.1111/1462-2920.13225 . 26842002

[39] Wang L, Liu K, Wang Z, Bai X, Peng Q, Jin L. Bacterial Community Diversity Associated With Different Utilization Efficiencies of Nitrogen in the Gastrointestinal Tract of Goats. Frontiers in microbiology. 2019;10:239. doi: 10.3389/fmicb.2019.00239. 30873128 PMC6401623

