# Supplementary material for: Oscillating dietary crude protein concentrations increase N retention of calves by affecting urea-N recycling and nitrogen metabolism of rumen bacteria and epithelium
Source: PLoS One. 2021 Sep 10;16(9):e0257417. doi: 10.1371/journal.pone.0257417 (PMC8432763; doi:10.1371/journal.pone.0257417)
Supplement: S1 Table — (DOCX) [file pone.0257417.s001.docx]

**S1 Table. Primers used for quantitative real-time PCR**

| **Gene name** | **Accession number** | **Primer sequence(5’→3’)** | **Product size (bp)** | **Annealing temperature (℃)** | **Amplification efficiency (%)** |
| --- | --- | --- | --- | --- | --- |
| ***β-actin*** | [NM_173979.3](https://www.ncbi.nlm.nih.gov/nucleotide/75832053?report=gbwithparts) | F: AGATCAAGATCATCGCGCCC | 172 | 60 | 100.1 |
|  |  | R: TAACGCAGCTAACAGTCCGC |  |  |  |
| ***CA14*** | [NM_001192205.1](https://www.ncbi.nlm.nih.gov/entrez/viewer.fcgi?db=nucleotide&id=300794535) | F: ATCGAAGGGCCCAGATTTCC | 161 | 60 | 99.8 |
|  |  | R: CGAGGGTCCCACTTGGATGA |  |  |  |
| ***CAT*** | NM_001035386.2 | F: TCACTCAGGTGCGGACTTTC | 162 | 60 | 101.8 |
|  |  | R: GGATGCGGGAGCCATATTCA |  |  |  |
| ***CYP11A1*** | NM_176644.2 | F: AGCTAGCATCAAGGAGACGC | 118 | 60 | 98.5 |
|  |  | R: CACTTGCACCAGTGTCTTGG |  |  |  |
| ***FMO5*** | NM_001101304.1 | F: CTCAGGTCTGGGTCTTGCAG | 101 | 60 | 98.4 |
|  |  | R: AACCTGTCTTTGAGGAAGCCA |  |  |  |
| ***GALE*** | NM_001206208.1 | F: GGAGCAGGACACACCAGTC | 198 | 60 | 100.2 |
|  |  | R: CAATGACCATGGGCGAATAGC |  |  |  |
| ***ALCAM*** | NM_174238.1 | F: AGGCAGACGAGATAAGTGATGA | 118 | 60 | 101.7 |
|  |  | R: AGCCAATAGACGACACCAGC |  |  |  |
| ***ALDOB*** | [NM_001034485.2](https://www.ncbi.nlm.nih.gov/entrez/viewer.fcgi?db=nucleotide&id=402745359) | F: GCCTCATCCACAGCTTCTGATA | 180 | 60 | 99.5 |
|  |  | R: ATCGTGCCTACGGACTCAT |  |  |  |
| ***CLCA1*** | NM_001206176.2 | F: GCAGCACAGGAACTAGAGCA | 152 | 60 | 99.7 |
|  |  | R: CTCTCAAGCTGAATGGAGCG |  |  |  |
| ***FBXO2*** | NM_001075569.1 | F: CCTTCGAGTGGTGTCGCAAA | 152 | 60 | 100 |
|  |  | R: CTTCACGGTGAGCTCGTACA |  |  |  |
| ***FZD4*** | NM_001206269.1 | F: GGGCTACAACGTGACCAAGA | 135 | 60 | 100.3 |
|  |  | R: ACGGAACAAAGGAAGAACTGC |  |  |  |
