# Supplementary material for: Oscillating dietary crude protein concentrations increase N retention of calves by affecting urea-N recycling and nitrogen metabolism of rumen bacteria and epithelium
Source: PLoS One. 2021 Sep 10;16(9):e0257417. doi: 10.1371/journal.pone.0257417 (PMC8432763; doi:10.1371/journal.pone.0257417)
Supplement: S2 Table — Gene ontology categories with corrected P values of enrichment significance below 0.05 are shown. (DOCX) [file pone.0257417.s002.docx]

**S2 Table. Gene ontology analysis of differentially expressed genes in liver**

| **Category** | **Gene ontology category (Accession No.)** | **Observed genes** | **Gene number** | **Enrichment score** | ***P* value** |
| --- | --- | --- | --- | --- | --- |
| **Biological process** | IRE1-mediated unfolded protein response (GO:0036498) | FKBP14; HYOU1; KDELR3; SERP1; WFS1 | 5 | 29.921 | <0.001 |
|  | ubiquitin-dependent ERAD pathway (GO:0030433) | DNAJB9; HSP90B1; HSPA5; WFS1 | 4 | 8.866 | <0.001 |
|  | response to hypoxia (GO:0001666) | EDNRA; EPO; HSP90B1; SLC11A2 | 4 | 7.365 | <0.001 |
|  | response to unfolded protein (GO:0006986) | DNAJB9; HSPA5; MANF | 3 | 9.974 | <0.001 |
|  | protein folding (GO:0006457) | CALR; DNAJB11; HSP90B1; MOGS | 4 | 5.503 | 0.001 |
|  | response to endoplasmic reticulum stress (GO:0034976) | DNAJB9; HYOU1; WFS1 | 3 | 5.984 | 0.002 |
|  | lipid metabolic process (GO:0006629) | LOC100138638; PLCXD1; SLC16A1 | 3 | 4.663 | 0.004 |
|  | translation (GO:0006412) | IGF2BP3; LOC506989; NHP2; RPL12 | 4 | 2.833 | 0.014 |
| **Cellular component** | endoplasmic reticulum chaperone complex (GO:0034663) | HSP90B1; HSPA5; HYOU1; SDF2L1 | 4 | 53.193 | <0.001 |
|  | endoplasmic reticulum lumen (GO:0005788) | DNAJB11; DNAJB9; FKBP14; HSP90B1; HSPA5; HYOU1; LOC516494; SDF2L1; WFS1 | 9 | 7.806 | <0.001 |
|  | endoplasmic reticulum (GO:0005783) | CCDC134; CRELD2; DNAJB9; GPR37; HM13; HSP90B1; HYOU1; KDELR3; LOC100848775; MAMDC2; MANF; MOGS; NUCB2; PRDX4; SEC23B; SERP1; WFS1 | 17 | 2.898 | <0.001 |
|  | endoplasmic reticulum membrane (GO:0005789) | FKBP2; GPR37; GUCY2C; HM13; HSP90B1; KDELR3; LOC100138638; LOC100848775; LOC527083; MOGS; RETSAT; SDF2L1; SEC23B; SEC61G; SERP1; TMEM178A; WFS1 | 17 | 2.806 | <0.001 |
|  | nucleolus (GO:0005730) | CDCA8; DNAJB9; DTL; EMG1; GNL3; METTL1; PPAN; REXO2; RPL12; SP100 | 10 | 1.912 | 0.016 |
|  | receptor complex (GO:0043235) | ACVR1C; GPR37; PRLR | 3 | 2.805 | 0.022 |
|  | extracellular space (GO:0005615) | A2ML1; APCS; CCL24; CRELD2; DNAJB11; EPO; EREG; GNL3; LOC100300442; LOC100336971; LOC781977; MANF; NUCB2; OBSCN; TAC1 | 15 | 1.616 | 0.023 |
|  | lysosome (GO:0005764) | LOC516494; LOC786974; MCOLN2; SLC11A2 | 4 | 2.430 | 0.025 |
|  | nuclear body (GO:0016604) | DTX1; GNL3; OBSCN; SP100 | 4 | 2.382 | 0.027 |
|  | Golgi apparatus (GO:0005794) | CRELD2; DUSP26; KDELR3; LOC100300442; LOC781977; MMGT1; NUCB2; SGSM1; SLC50A1; SYNDIG1L | 10 | 1.742 | 0.029 |
| **Molecular function** | peptidyl-prolyl cis-trans isomerase activity (GO:0003755) | FKBP11; FKBP14; FKBP2 | 3 | 9.974 | <0.001 |
|  | rRNA binding (GO:0019843) | EMG1; PPAN; RPL12 | 3 | 9.207 | <0.001 |
|  | unfolded protein binding (GO:0051082) | CALR; DNAJB11; HSP90B1; HSPA5 | 4 | 5.148 | 0.001 |
|  | cytokine activity (GO:0005125) | EPO; LOC100300442; LOC100336971; LOC781977 | 4 | 2.720 | 0.016 |
|  | iron ion binding (GO:0005506) | CYP11A1; LOC527083; SLC11A2 | 3 | 2.872 | 0.021 |
|  | ubiquitin protein ligase binding (GO:0031625) | DTX1; GPR37; HM13; WFS1 | 4 | 2.546 | 0.021 |
|  | growth factor activity (GO:0008083) | EREG; LOC100336971; MANF | 3 | 2.827 | 0.022 |
|  | carbohydrate binding (GO:0030246) | APCS; CALR; KLRC1 | 3 | 2.621 | 0.028 |
|  | protein homodimerization activity (GO:0042803) | CDH13; GALE; HM13; LOC100848775; LOC519737; MGAT2; NR4A1; PRDX4; SP100 | 9 | 1.778 | 0.031 |

Gene ontology categories with corrected *P* values of enrichment significance below 0.05 are shown.
